# Biological strategies in rotator cuff repair: a clinical application and molecular background

**DOI:** 10.1530/EOR-24-0012

**Published:** 2024-12-02

**Authors:** Maciej Pawlak, Joanna Wałecka, Przemysław Lubiatowski

**Affiliations:** 1 Rehasport Clinic, Poznań-Gdańsk, Poland; 2 Sport Traumatology and Biomechanics Unit, Department of Traumatology, Orthopaedics and Hand Surgery, Poznań University of Medical Science, Poznań, Poland

**Keywords:** rotator cuff repair, biological therapy, platelet-rich plasma, stem cells, bone marrow-derived stem cells, adipocyte-derived stem cells, biological scaffolds

## Abstract

- Conventional repair of rotator cuff tears bears a variable but significant risk of incomplete healing.
- Biological therapies that accompany surgical rotator cuff repair include platelet-rich plasma, stem cells of different origins, and biological scaffolds.
- Biological therapies facilitate the regeneration of the correct microarchitecture of the tendon attachment to the bone and reduce failures after surgical rotator cuff repair.

## Introduction

Rotator cuff (RC) tears are a widespread medical problem, with a 20% prevalence in the general population (1, 2). RC repair is indicated based on a combination of patient symptoms, physical examination findings, imaging results, and the patient's functional needs and expectations. Barkatali and Collins, in their recent publication, emphasize a patient-centered approach, considering individual symptoms, functional needs, and overall health status (3). The primary indications include large-to-massive RC tears (4). Other signs (relative indications) that RC surgery should be considered include: symptoms lasting 6–12 months, large tears (more than 3 cm) with good quality of the surrounding tendon tissue, significant weakness and loss of shoulder function, muscle atrophy and fatty infiltration seen on imaging studies, a recent acute injury, or failure of non-surgical management after 3–6 months (4). Despite the general clinical success of the surgical approach and multiple improvements, the failure rates of healing are still high and might account for 8% (as reported by Snyder group in 2021 (5)), 19% (6), 43% (7), or even up to 90% in elderly patients (8). The effectiveness of healing after RC repair is variable and influenced by multiple factors (8, 9, 10). Moreover, RC repair cannot restore normal complex structure of the tendon-to-bone junction (11) and, at most, results in fibro-vascular scar tissue formation (rich in collagen III (12, 13)). Inferior structure and persistent tissue defects that remain after arthroscopic RC repair reduce tendon biomechanical properties (8, 14, 15, 16). Among others, poor blood supply has been pinpointed as one of the mechanisms implicated in altered tendon healing (12, 17, 18, 19). Multiple other factors of failed healing after surgical repair have been identified, e.g. intrinsic tendon degeneration, fatty infiltration of tendon and muscle, and muscle atrophy (20).

The hypothesis that biological therapies might facilitate the regeneration of the normal tendon-to-bone insertion microarchitecture and limit the amount of scar tissue has been postulated for some time now (21, 22). It has been shown, mostly in laboratory settings and to some extent in clinical scenarios, that better repair effects through external implementation of growth factor and/or stem cell therapy (presented in the next chapters) during tendon healing can be achieved. Nevertheless, new biological approaches are required to improve tendon healing. There are several reviews concerning the biological methods that might improve the healing of freshly operated RC. However, they do not precisely separate the methods that are currently used in everyday surgical practice from the methods that might be good alternative in the future. Moreover, multiple reviews discuss the biological factors without presenting the information about the form of their delivery to the healing site. We have therefore focused on systematizing the existing knowledge on clinical strategies, and in the current review, we are presenting two points of view: the clinical – which biological methods assist the surgical repair of the RC, and the biological – what are the underlying molecular mechanisms of action.

We have taken into consideration only those therapies that have clinical application in humans and whose results have been published. This includes platelet-rich plasma (PRP), stem cells, and biological scaffolds.

### Platelet-rich plasma

PRP, also known as platelet-derived growth factors (PDGFs), platelet-rich fibrin (PRF), and platelet concentrate, is a biological product defined as a portion of the plasma of autologous blood with high platelet concentration. A concentration of approximately 1 500 000/μL (1.5 mln of platelets per 1 μL of plasma) has been suggested as the working definition of PRP (23). This is a platelet count five to eight times higher than that of blood.

**Figure 1:**
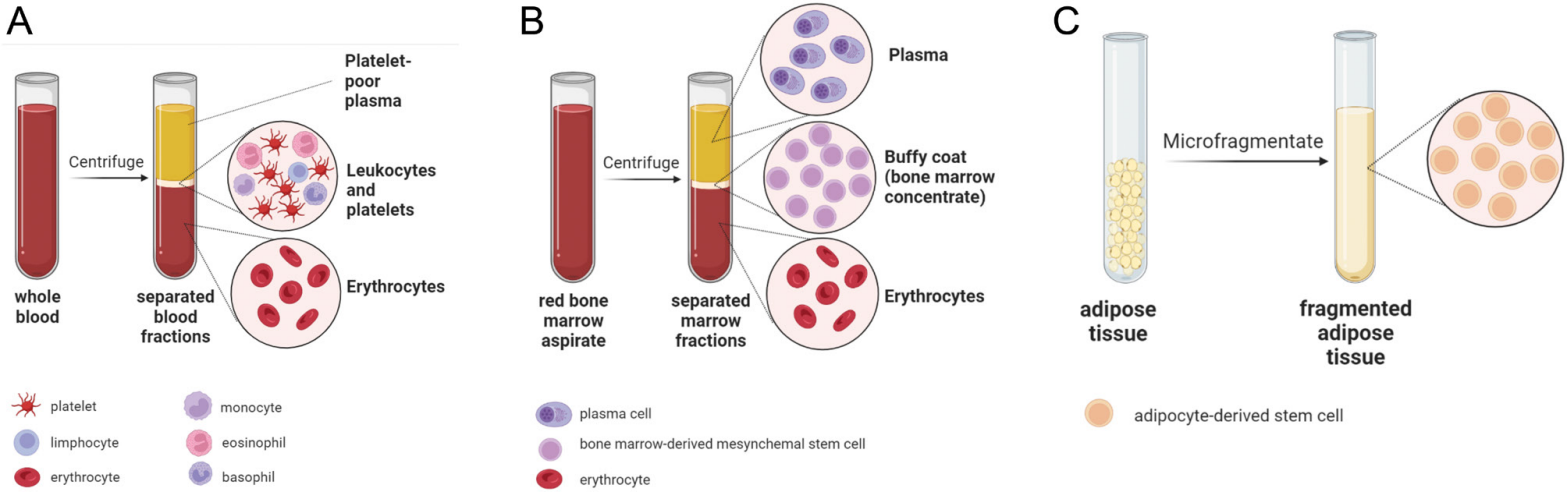
Preparation of the platelet-rich plasma from the whole blood (A), the bone marrow concentrate, as a source of bone marrow-derived mesenchymal stem cells (B), and fragmented autologous adipose tissue, as a source of adipose-derived mesenchymal stem cells (C).

A variety of available commercial systems for PRP production, each of which operates through different techniques and yields varying platelet concentrations, are currently available. However, most commonly, the PRP is obtained from the venous blood (typically 10 mL) of patients by centrifugation, during which the separation of the blood components follows according to their different density gradients. After centrifugation, the end product is visible as three layers (Fig. 1A): the bottom-red layer (erythrocytes), the middle-white layer (leukocytes, inflammatory cytokines, platelets), and the yellow-top layer (platelet-poor plasma). The PRP layer (the thin layer between the middle and the top layers) is then collected, characterized (the platelet concentration is measured using a hematology analyzer), and injected into the desired site.

Platelets (thrombocytes) are the end products of megakaryocytes and are formed in the bone marrow. They lack a nucleus and cannot replicate; thus, the lifespan of a platelet is 5–9 days. They are the smallest blood cells, approximately 2 μm in diameter. They contain organelles and structures such as mitochondria, microtubules, and granules (α, δ, λ). The α granules in the platelets contain more than 30 bioactive proteins, many of which have a fundamental role in hemostasis and/or tissue healing (24). These proteins include growth factors, chemokines, cytokines, and other plasma proteins (25).

After a tissue injury or a surgical stimulus, when platelets become exposed to damaged blood vessels, the platelets aggregate at the site. This process is called activation. During activation, the α granules release some of their protein contents (including growth factors) to the surroundings (degranulation). The platelets begin actively secreting these proteins within 10 min after clotting, with more than 95% of the growth factors secreted within 1 h (24). After this initial burst of PRP-related growth factors, the platelets synthesize and secrete additional growth factors for the remaining several days of their lifespan.

The regenerative potential of PRP depends on the amount of growth factors released when the platelets are activated. The main growth factors present in the platelets include platelet-derived growth factor (PDGF), transforming growth factor-beta (TGF-ß), and vascular endothelial growth factor (VEGF). The main function of PDGF is stimulating cellular replication (mitogenesis) and promoting angiogenesis (26). Thus, PDGF is considered a key regulatory factor in tissue repair and regeneration. This growth factor increases cell populations of the healing cells, including mesenchymal stem cells (MSCs) and osteoprogenitor cells. Therefore, PDGF can also enhance stem cell-based bone regeneration (discussed further in the next section). TGF-ß modulates bone matrix synthesis by increasing the number of cells capable of expressing osteoblasts and decreases bone resorption by inducing the apoptosis of osteoclasts (27). In addition, TGF-ß activates fibroblasts to induce collagen formation, endothelial cells for angiogenesis, chondroprogenitor cells for cartilage, and mesenchymal cells to increase the population of wound healing cells. VEGF is a subfamily of a PDGF family of cystine-knot growth factors. They are important signaling proteins involved in both vasculogenesis and angiogenesis, which pave the way for healing (27).

**Figure 2:**
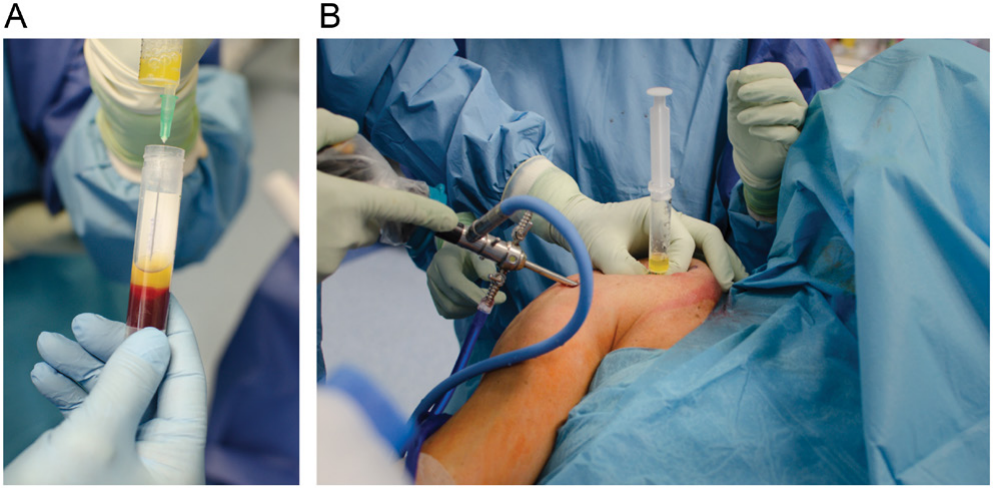
Administration of the platelet-rich plasma in the surgical setting. Aspiration of the PRP fraction (A), and administration to the traumatized area during rotator cuff repair surgery (B).

When performing RC repair, PRP is administered in the surgical setting so that growth factors released by the platelets may recruit reparative cells and assist with the augmentation of the surgical repair (Fig. 2). Over the past decade, there has been increased attention to PRP in the setting of RC repair. From the clinical perspective, an important issue is the procedure and the form of PRP application, as well as its administration site. The PRP administration schedule varies in different studies. PRP can be used as a single administration during surgery (28, 29, 30, 31) or additionally during follow-up visits, e.g. once a week for 4 weeks (31, 32). PRP can be solid (30) or liquid (28, 31, 32, 33). Activation of leukocyte-reduced PRP with autologous clotting factors using an autologous activation system (28) or autologous thrombin and calcium chloride (33) has been used in some units. Many studies do not provide information on the molecular parameters of the PRP (including platelet concentration), which is surprising given the expected correlation of application results with the number of platelets administered. Malavolta *et al.* found that their PRP preparation method (liquid form, PRP liquid was mixed with pre-prepared autologous thrombin and calcium chloride) resulted in an average platelet concentration of 1 185 166/mm^3^, which is a 7.65-fold increase compared to the output (33), and this is consistent with the working definition of PRP (23).

The most comprehensive evaluation of the efficacy of PRP in augmenting RC repair is found in meta-analyses of prospective trials. Clinical results in several publications have often been often contradictory and inconclusive regarding the clinical efficacy of PRP. Yet most recently, Ahmad *et al. (* 34) conducted a systematic review of meta-analyses of RC repair using PRP to identify that PRP improves clinical function and decreases the rate of tendon re-tears. They examined 13 meta-analysis articles published up to November 2019. The initial studies published between 2012 and 2015 suggested that PRP for RC tendinopathy/impingement syndrome (non-operative treatment) is NOT effective (34). However, all of these studies were inadequately powered (they included under 500 patients, presented insufficient clinical evidence or an insufficient number of randomized controlled studies). With the increasing amount of patient data, later meta-analyses were able to detect reduced tendon re-tear rates, improved pain scores, and functional outcomes in all types of tears. Warth *et al.* first noted in 2015 the possibility of reducing re-tear rates after PRP application (35). In this study, the authors decided to undertake a subgroup analysis by tear size (the initial tear size of <3 cm or >3 cm sagittal length) and, as a result, were the first to find PRP effective for small and medium tears. Articles by Zhang *et al*. (36), Vavken *et al*. (37), and Cai *et al*. (38) echoed this. However, when cuff tears greater than 3 cm in anterior–posterior length were repaired using a double-row technique, a statistically significant reduction in re-tear rates was also noted (35).

The PRP effectiveness was also evaluated in meta-analyses in terms of the PRP preparation and application method since different studies reported varying PRP preparation protocols (28, 29, 30, 31, 32, 33) that may have produced PRP solutions with variable platelet concentrations, biochemical activation cascades, and leukocyte concentrations. Neither different PRP preparation methods (manual vs commercially available PRP preparation systems) nor the PRP form (fibrin matrix vs liquid) resulted in a change in the overall effect of PRP treatment on re-tear rates (34, 35). However, the exact place of PRP application to the repaired tendon (PRP injection into the arthroscopic working portal after RC repair vs placing PRP at the tendon–bone interface where healing occurs) has an impact on the outcome of treatment. The overall gain in the constant score (CS) was observed when PRP was administered at the tendon–bone interface rather than over the surface of the repaired tendon (34, 35).

A number of studies tackled the point of the leukocyte content in PRP, and a recent meta-analysis showed common consensus with the conclusion that better results are observed with the use of leukocyte-poor PRP than with the use of leukocyte-rich PRP (39). The meta-analysis showed that leukocyte-poor PRP significantly reduced the re-tear rate in RC repair, and improved clinical results. In contrast, the efficacy of leukocyte-rich PRP was not significantly improved with the exception of the visual analogue scale (VAS) score. We would therefore recommend using leukocyte-poor PRP as a biological enhancement of RC surgical repair.

Another important issue worth discussing here is that the RC undergoes various tendinopathic and avascular changes during the aging process, altering its healing capacity and increasing failure rates after surgical treatment. Since PRP treatment improves tendon healing, many studies have focused on using PRP for the treatment of RC tendinopathy. In 2017, Miranda *et al.* performed a systematic review of current evidence supporting the use of PRP in RC pathologies (40). It appeared that although most of the preclinical studies report positive results for PRP in RC tendinopathy, the same success is not translated into clinical settings – 70% of clinical studies suggest minimal to no difference between the PRP group and the control group. However, other studies have suggested both short-term and long-term pain reduction and functional improvement with a single PRP injection into the RC (reviewed, e.g. in refs. (41, 42)). The general conclusion from these studies is, therefore, that although PRP therapy produces significant long-term pain relief, it does not show significant differences in functional results. PRP may therefore be a promising treatment option for RC tendinopathy, but more consistent evidence is still needed.

The benefits of the PRP application after RC repair are now encouraging; however, the application of PRP, as well as similar biological products, in an attempt to enhance the biological healing process is associated with significant costs. Samuelson *et al*. performed a cost-utility analysis to determine if the use of PRP products during arthroscopic RC repair is cost-effective (43). This cost-utility study, using the most current literature, showed that the augmentation of RC repairs with PRP is not cost-effective. The cost of PRP application is assumed to be around $750–$1000; therefore, the additional use of PRP in arthroscopy would need to be associated with a reduction in the re-tear rate of approximately 9% before its use would be considered cost-effective. The most recent data assume the possible re-tear rate is found to be 15% within 3 months after surgery, 16% at 6–12 months follow-up, and at follow-up longer than 24  months, 21% at 3–6 months, and 12–24 months follow-up (44).

### Stem cells

Stem cells, either embryonic or adult stem cells, are undifferentiated cells that have the potential to differentiate into certain adult cell types of mesenchymal origin (i.e. bone, fat, tendon, muscle, and cartilage) and proliferate indefinitely (45). Formulations containing such connective tissue progenitors, including MSCs (stromal), are very promising tools in tissue engineering, orthopedic surgery, and regenerative medicine, showing outstanding therapeutic efficacy (46, 47). The application of preparations rich in MSCs to enhance RC tendon healing and to manage musculoskeletal shoulder disorders has gained ground due to their potential to differentiate towards different target cells and their anti-inflammatory and angiogenic characteristics. However, one has to keep in mind that based on the current state of the art, there is very little data to suggest that implanted cells undergo differentiation into other cell types. Moreover, the current concept of stem cell function includes a paracrine mechanism and the production of numerous signaling molecules that have an important effect on healing, largely through anti-inflammatory and immune-modulatory mechanisms (48). Different tissue sources for MSCs have been identified, including bone marrow, adipose tissue, muscles, synovia, periosteum, tendon, dermis, and umbilical cord, or peripheral blood, and have been evaluated as sources of multipotent and pluripotent cells (49). The main types of adult MSCs are (i) bone marrow-derived MSCs (BM-MSCs), (ii) adipose-derived MSCs (A-MSCs), (iii) tenocyte-derived MSCs (T-MSCs), (iv) umbilical cord-derived MSCs, (v) urine-derived MSCs, and (vi) bursa-derived MSCs (BD-MSCs).

### Bone marrow-derived mesenchymal stem cells

BM-MSCs, the first-discovered MSCs, are pluripotent cells with multilineage differentiation ability into adipocytes, osteoblasts, chondrocytes, and tenocytes. Bone marrow containing connective tissue progenitors, growth factors, and other elements from the locally drilled holes is recruited into the repaired tendon to accelerate tendon and bone healing. The BM-MSCs-rich formulations used in RC injury could be also harvested, mainly from the iliac crest or proximal humerus bone marrow, and administered as an injection of bone marrow concentrate (BMC).

#### Bone marrow stimulation

**Figure 3:**
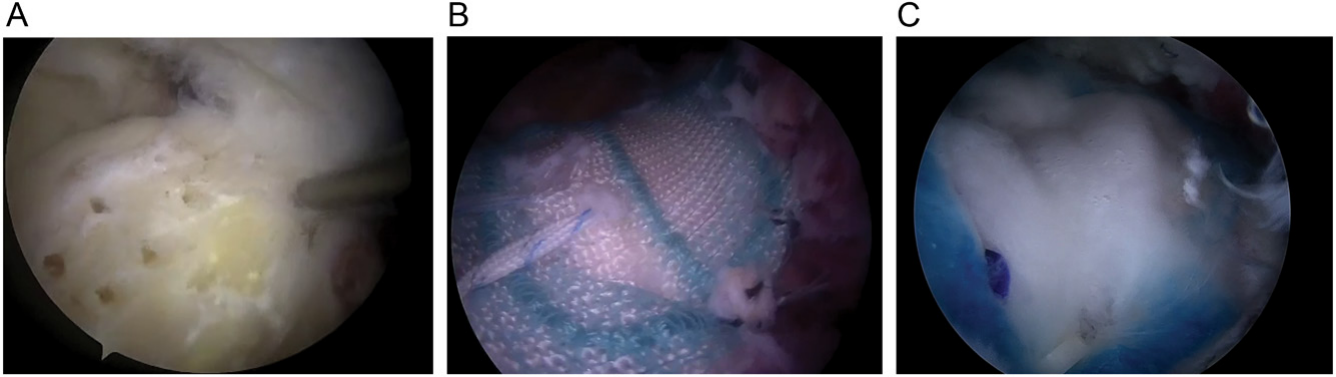
The appearance of microfractures (A), synthetic (B), and biological (C) patches during rotator cuff surgery. The membranes are fixed using tendon (marked in violet) and bone anchors (marked in gray).

The model of providing bone marrow-derived connective tissue progenitors to the healing tendon-to-bone area of the RC through bone marrow stimulation typically utilizes microfractures or multiple channeling techniques at the footprint area (Fig. 3A). An abrasion arthroplasty could also be the source of bone marrow stimulation; however, since it requires a complete removal of about 1 mm of the subchondral bone, it may increase the pull-out risk of the anchors.

A variety of available instruments were presented, including arthroscopic awls (50), metal bars (51), or mechanical bars (52) to create microfractures at different locations of the injury site (53). The intervention during microfractures involves drilling the perforations 3–4 mm apart and 2–5 mm deep. One could therefore assume that deepening the channels would benefit in better delivery of marrow elements. Therefore, a deep marrow venting via multiple channeling techniques was proposed, which relies on creating several bone vents on the footprint area (the RC tendon in the greater tuberosity of the proximal humerus), a ‘crimson duvet,’ which contains abundant marrow MSCs.

The bone marrow stimulation techniques are more commonly used to support the healing of the RC than the injections of BMC. Already in 2013, Osti *et al.* reported that patients undergoing RC repair and microfractures of the bone at the footprint of the RC fare better in University of California-Los Angeles (UCLA) score, CS, pain, and range of motion at 3 months than patients undergoing RC repair only (54). Since that time, many studies focused on marrow stimulation and reported high rates of RC healing (55, 56, 57). A very recent publication gathered the results of nine systematic reviews and meta-analyses of bone marrow stimulation-oriented studies from 2010 to 2022 (58). The studies comprised 756 patients. Single-row repair techniques were performed in 527 (72.2%) patients, and double-row techniques – in 203 patients (27.8%). The pooled re-tear rate across all cited studies for patients undergoing marrow stimulation was only 11%, and the reported Constant score was significantly higher in the marrow stimulation group.

Two recent meta-analyses supported the advantages of bone marrow stimulation in arthroscopic RC repair and additionally presented the cost-effective advantages (53, 59). Bone marrow stimulation is a straightforward technique that does not require additional costs or particular instruments, and it can be completed in approximately 10 min.

#### Bone marrow concentrate

A variety of approved commercial systems for BMC production are available (a comparison of some systems is presented in ref. (60)). All these systems operate through similar techniques and are based on two major steps (61): (i) bone marrow collection and (ii) concentration by centrifugation. Briefly, the procedure of BMC isolation takes place during RC repair (typically shortly after the procedure), under general anesthesia. Several skin and bone punctures are required to extract sufficient bone marrow. Typically, a volume of 150 mL of marrow is aspirated from the anterior iliac crest in small fractions of ~4 mL to reduce the degree of dilution by the peripheral blood (no more than 3–4 mL of a bone marrow should be aspirated, as further aspiration will simply yield peripheral blood). All aspirates are further combined and concentrated by centrifugation to produce BMC. After centrifugation, the end product is visible as three layers (Fig. 1B): the bottom, red layer (erythrocytes), the middle, buffy layer (BMC), and the top layer (plasma). The buffy coat containing MSCs is then collected, a small portion is secured for laboratory testing, and the rest is injected into the desired site, usually at the end of RC tendon fixation, between the bone and tendon or in the bone. The laboratory testing includes culturing MSCs under *in vitro* culture conditions and measuring their concentration using a cell counter.

To date, several reports have described the effect of BMC injections in acute tears and repairs of the supraspinatus tendon, with the majority of those studies performed on rats, but also including one dog and a rabbit. Gulotta *et al*. performed a series of studies testing the influence of unmodified and modified BM-MSCs-containing concentrate (62, 63, 64) on RC repair. In the first study, BMC isolated from rats’ long bones were injected on the repair side; however, the cells failed to improve the structural and biomechanical aspects of healing (62). Importantly, genetic modification of MSCs to overexpress the developmental gene membrane type 1 matrix metalloproteinase (MT1-MMP) increased structural and material biomechanical properties and elevated the amount of fibrocartilage at the tendon-to-bone interface (63). Improvements in histological cartilage formation at the injection site and enhancement of biomechanical properties have been achieved by modifying MSCs with scleraxis, a transcription factor believed to direct the tendinous attachments to bone (64). This research on scleraxis has shown promise; however, the apparent lack of recent studies on this topic suggests that it has lost its popularity, which may be attributed to several factors. Among them, ensuring the safety and efficacy of genetically modified cells, as well as the emergence of novel technologies such as gene editing, exosome therapy, or bioprinting) is a significant concern.

Despite extensive animal experimental studies, there are only a few clinical studies evaluating the clinical efficacy and safety of using injections of BMC in RC tear repair in humans. Hernigou et al. (65) demonstrated that the injection of BMC into the tendon-to-bone interface as an adjunctive therapy enhanced the healing rates and prevented further tears in patients after the RC repair. Significant improvement in healing outcomes at the 10-year follow-up, with 87% of patients in the BMC-treatment group demonstrating tendon integrity compared to just 44% of control group patients, was achieved. Furthermore, Hernigou *et al.* showed a substantial improvement in the level of tendon integrity. In another clinical study, 14 patients with complete tears of the RCs underwent repairs with sutures and augmentation with mononuclear stem cells from the iliac crest bone marrow (66). After 12 months, in all patients, the tears had healed according to clinical and MRI criteria, and 13 out of 14 patients had improved clinical outcomes compared with historical data available for patients undergoing the same surgical procedure without the addition of stem cells. Although the above-mentioned studies demonstrated that BMC injections promote RC repair healing, only one randomized prospective study investigating its clinical efficacy has been published so far (67). The study by Cole *et al*. presented the analysis of patients undergoing RC repair for isolated 1–3-cm supraspinatus tendon tears with BMC augmentation (a control group underwent sham incision). Functional outcomes, measured using the American Shoulder and Elbow Surgeons (ASES), Single Assessment Numeric Evaluation (SANE), Simple Shoulder Test, 12-Item Short Form Health Survey, and Veterans RAND 12-Item Health Survey revealed significant improvements in functional indices by 6 months, maintained at 1 and 2 years. Moreover, MRI at 1-year follow-up demonstrated a significant improvement in the structural integrity of the tendon. However, BMC treatment did not lead to a meaningful enhancement in clinical outcomes or lower treatment failure rates compared to the RCR alone. The authors left the readers with the conclusion that further research is necessary to look into the long-term implications of improved repair quality on surgical success and patient outcomes.

### Adipose-derived stem cells

Adipose-derived stromal cells (A-MSCs) can be isolated in large quantities from subcutaneous adipose tissue and liposuction aspirates (68). The procedure of obtaining a preparation rich in A-MSCs takes place during RC repair, under general anesthesia. Adipose tissue (approximately 100 mL) is first collected by needle biopsy or liposuction aspiration. The harvested fat is immediately processed mechanically to reduce the size of the adipose tissue clusters and eliminate blood residues with pro-inflammatory properties during constant irrigation. The resulting micro-fragmented tissue (Fig. 1C) is collected and re-injected into the desired site. Several adipose tissue dissociation devices are available. Another possibility to obtain a preparation rich in A-MSCs is the stromal vascular fraction (SVF), a heterogeneous collection of cells contained within adipose tissue that is traditionally isolated using enzymes such as collagenase. SVF production requires enzymatic digestion, and currently, the process is only FDA-approved in the United States. Therefore, the regenerative cell therapy based on SVF is at an early investigative stage, but its potential for clinical application is enormous.

Due to A-MSCs' high accessibility and their potential to differentiate into tenocytes (tendon cells), they are a promising source of stem cells in regeneration therapy. Using formulations rich in A-MSCs to improve RC repair is a viable option because of their easy assimilation and the ability to inhibit osteogenic differentiation through microenvironmental modulation and anti-inflammatory properties (69, 70). Local administration of connective tissue progenitors, including ADSCs, into the musculotendinous junction area of the subscapularis in rabbits resulted in an improvement in muscle function, tendon healing, and a decrease in fatty infiltration after cuff repair (71). Furthermore, A-MSCs mediated acute inflammation with diminished presence of edema and neutrophils, but biomechanical properties of tendon–bone healing from 2 to 8 weeks after repair in a rat acute RC repair model were not improved (72). Significant improvement at a minimum of 12 months of structural outcomes assessed in terms of the retear rate and MRI results (where the re-tear rate of the A-MSCs group (14.3%) was less than that of the control group (28.5%)) was recorded for patients treated with an injection of ADSCs loaded in fibrin glue during arthroscopic RC repair (73).

An improvement in shoulder function scores and RC strength for up to 2 years post-treatment after intra-tendinous injection of preparations rich in A-MSCs in patients with partial-thickness rotator cuff tears (sPTRCT) has been reported in a recent study by Jo *et al.* (74). The bursal-sided defects almost disappeared at 1 year and did not recur for up to 2 years. In another study (75) a single injection of unmodified, autologous adipose-derived regenerative cells (UA-ADRC) in patients with sPTRCT who did not respond to physical therapy treatments showed significantly higher total scores on the ASES at weeks 24 and 52 post treatment.

The therapeutic effects of ADSCs may be attributed to the mediation of acute inflammation, with the diminished presence of edema and neutrophils (76), reversing the dominated fibrovascular scar response in acute tendon–bone healing (77), increasing bone mineral density of the proximal humerus to promote tendon–bone healing in repairs of chronic tears (78), improving muscle function and decreasing fatty infiltration of the muscle, enhancing the load-to-failure in chronic RC tears (67), and increasing fibrocartilage area, bone volume/total volume values, and biomechanical properties (78, 79). The role of the adipose tissue-derived cells in enhancing the healing of RC injuries by activating resident human tendon stem cells *in situ* was studied in detail by Randelli *et al.* (80). The authors measured tendon stem cell proliferation rates, morphological changes, expression of stem cell markers, levels of VEGF, and assessed the differentiation and migration abilities of the cells after their treatment with microfragmented adipose tissue. The results indicated increased proliferation of the stem cells, proper differentiation and migration capabilities, as well as a notable increase in the expression of VEGF in the treated cells, highlighting its potential role in promoting neovascularization, which is essential for tissue healing.

### Bursa-derived MSCs

The discovery of resident bursa-derived cells' stem cell potential expounds on the functions of subacromial bursa, as it can augment the regenerative properties of adjacent tissues such as RC tendons (81). The MSCs isolated from the subacromial bursa tissues were characterized for osteoblastic, adipogenic, chondrogenic, and tenogenic differentiation *in vitro* and *in vivo*, with high proliferative capacity, and differentiated toward cells of mesenchymal lineages with high efficiency (82). Furthermore, when treated with bone morphogenetic protein-12 (BMP-12), the cells expressed markers of tenocytes and assumed aligned morphology in culture, and when seeded in ceramic scaffolds, formed extensive bone, as well as tendon-like tissue *in vivo* (81). It has been postulated that several BD-MSCs qualities, such as the tight fibrovascular network, a high growth factor content, and the large progenitor potential of bursa-derived cells, could augment the RC healing process (82). These hypotheses have been proven in mice implanted with BD-MSCs that were subjected to BMP-12, seeded onto ceramic scaffolds, and observed as *in vitro* multipotent differentiation and extensive bone and tendon-like tissue formation. Moreover, superior engraftment into the host tissue and greater cell survival using BD-MSCs that were incorporated into fibrin gels have been reported in a patella tendon defect model in mice (83).

So far, there is only one study aimed at comparing the cellular viability and differentiation potential of subacromial bursa-derived cell augmentation to the RC in patients undergoing primary and revision arthroscopic RC repair (84). This study included 30 patients. The most important finding of the study was that subacromial bursa-derived cells located over the RC muscle and tendon of patients are viable and possess a differentiation potential for cartilage, bone, and adipose formation. Moreover, there were significantly more colony-forming units of MSCs derived from the subacromial bursa over the RC muscle in patients undergoing primary repairs compared with revision. These findings demonstrate that subacromial BD-MSCs may be used for augmentation in the challenging treatment of recurrent RC tears.

In conclusion, only the utilization of bone marrow stimulation as a source of connective tissue progenitors has been well-proven in the treatment of RC injuries. While alternative cell sources remain promising, there is still very limited data available. Importantly, the current concept is that cell therapy formulations work via a paracrine mechanism, by the production of various cytokines and other signaling molecules.

What needs to be strengthened here is that although there is increasing evidence that administered formulations containing MSCs can positively influence the regeneration of damaged tissues, the fate of the transplanted cells is still under debate. Most studies agree that when formulations rich in MSCs are IA-injected, cells tend to remain at the injection site, because the joint provides a confined space, such as observed in the knees of rabbit models (85). However, in another study, MSCs were found in the knee cavity and synovial tissue 1 day after injection, but could not be detected in the knee joint 1 week post-injection (86). The data to prove that injected cells engraft into the injected area in humans are still missing.

### Augmentation with scaffolds

To reconstruct the RC and to optimize tendon healing, scaffolds (also named patches) can be used to bridge gaps, augment RC repair, and enhance healing potential. The biological function of scaffolds is either to provide initial mechanical augmentation to the repaired cuff, absorbing part of the stress and thereby protecting the suture (e.g. human decellularized dermal matrix grafts) or to support new tissue formation (e.g. Regeneten implant). Two types of scaffolds are currently in use: synthetic and biological ones (reviewed in ref. (87)) (Fig. 3B and C). They differ in the type of materials used. Synthetic patches can be produced from a variety of polymers, with the most popular being polytetrafluoroethylene (teflon) grafts, first reported in 1986 (88).

**Figure 4:**
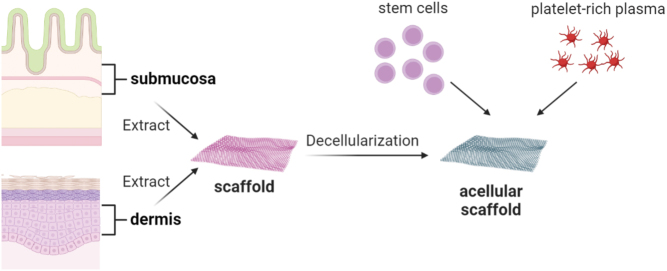
Preparation of acellular biological scaffolds from small intestine submucosa or dermis, with optional augmentation with stem cells and/or platelet-rich plasma.

The biological scaffolds can used as autografts or produced from xenografts or allografts by the removal of cellular components to produce a decellularized extracellular matrix (ECM), which minimizes the potential problem of graft rejection. Autografts have the major advantage of no risk of immunologic reaction and no extra cost. The best example is long head of the biceps, which can be used as augmentation of repair or even the equivalent of superior capsular reconstruction (89, 90, 91). A major limitation is the potential lack of tissue due to degeneration or previous rupture. Another autologous material is fascia lata, which offers good quality tissue that can be folded if needed (92). A major disadvantage is the necessity of harvesting (operating time, donor site morbidity). The most widely used xenografts include porcine-derived membranes from the small intestine submucosa or dermis. The allografts are produced from dermal skin grafts (which comprise the epidermis and upper part of the dermis) and are retrieved from deceased tissue donors. In all biological membranes, the crucial step is complete decellularization (Fig. 4), which could be maintained by three main approaches: physical (snap freezing or mechanical agitation), chemical (lysis of native tissue cells with hypotonic solutions or detergents followed by the solubilization and removal of the cellular remnants by washing), and enzymatic (trypsin). The importance of decellularization might be strengthened by the fact that the Restore membranes are now not recommended for use because they were found to contain a relatively high level of DNA, which resulted in an inflammatory response in patients (87, 93, 94).

The biological function of scaffolds is to provide initial mechanical augmentation to the repaired cuff, absorbing part of the stress and thereby protecting the suture. The healing between the repaired RC tendon and bone is dependent on bone ingrowth (95). Because of the importance of bone ingrowth in tendon-to-bone healing and the fact that RC tendon healing is often limited by deficient tissue formation with gap formation in the surgical site, the biological scaffolds could improve the healing of a tendon attached to the bone surface. At the same time, the biological scaffold is a good ground for host cell colonization, leading to the formation of a tendon structure with similar histological and mechanical features to the native one, but with greater thickness. The biological patches, despite their origin, might be of different sizes and thicknesses (adjusted accordingly to the wound size), but they are all made mostly of collagen, which is responsible for the improvement in the healing of the surgery site. Therefore, to provide resistance to collagenase enzymes responsible for the breakdown and resorption of implanted collagen, a cross-linking approach might be used. Moreover, after implantation, the patch can allow local delivery of growth factors, support fibroblast infiltration and revascularization so that it is gradually incorporated into the surrounding tissue, thus providing strength and support while inhibiting wound contraction through scarring.

Another important value of the biological collagen scaffolds is the fact that they can be additionally supported with other biological agents, like PRP or stem cell extracts, as described in previous paragraphs. Concerning that, the biological scaffolds can be treated as cell-based tissue engineering approaches.

Most recently, there has been the emergence of a highly processed biological scaffold, potentially meriting a new type of scaffold category. A highly porous and purified absorbable bovine collagen scaffold, which induces biological repair while having no mechanical support to the repair construct, has been presented, and so far, at least 12 published clinical studies including 709 patients have reported the outcomes of this strategy. These bioinducible implants have been shown to support healing of sPTRCT, maintaining the integrity of the repair for 2 years (96) and 5 years (97), but also of large and massive RC tears, with equal treatment success between primary and revision groups 2 years after the treatment (98). Importantly, McIntyre *et al.* collected patient-reported outcomes from 173 patients treated arthroscopically with the application of a bioinductive implant in 15 different centers located in the Unites States (99). Partial- and full-thickness RC tears showed improved patient-reported outcomes at 1-year follow-up: in the partial-thickness group, 84% and 83% of patients reported improvement in the VAS pain and ASES scores, respectively, and in the full-thickness group, 72% and 77% of the patients improved their scores in VAS pain and ASES, respectively.

Recent systematic reviews have looked at healing rates for various scaffolds in augmentation, e.g. refs. (91), (92), (99), (100), (101), and (102). All of them indicated that using biological scaffolds improved the clinical outcomes of RC tendon–bone repair. Moreover, in 2019, Bailey *et al.* performed a systematic review of 36 publications and a meta-analysis of five comparative publications (with a total of 397 cases) on the outcomes of graft augmentation or interposition vs RC repair alone (103). This meta-analysis evaluated whether the use of a graft leads to superior outcomes vs RCR alone. The authors concluded that graft augmentation or interposition appeared to provide a lower re-tear rate and improved ASES scores when compared with RC repair alone. One year later, Baldwin *et al.* screened 60 publications in their systematic review and performed a meta-analysis of 20 comparative publications (with a total of 1128 cases) (104). So far, it is the largest systematic appraisal of the clinical effectiveness and safety of implantable meshes for augmented RC repair. This meta-analysis suggested a small improvement in pain and patient-reported outcome measures for synthetic patches, and a moderate reduction in re-tear rate for synthetic and allograft patches.

Moreover, a recent survey of surgeon members of the British Elbow and Shoulder Society (BESS) revealed that 58% of respondents had used a patch to augment RC surgery (105). Most of the patches used were constructed from human decellularized dermis tissue, although porcine-derived and synthetic-based patches had also been used.

## Conclusion

RC injuries lead to persistent symptoms and significantly diminish function and quality of life. Unfortunately, the clinical capabilities of surgery and conventional therapies for the treatment of RC injuries still remain imperfect from a biological healing perspective. Several studies show the facilitation of the regeneration of the correct tendon-to-bone insertion microarchitecture and the limitation of the amount of scar tissue with the use of biological therapies that include PRP (with growth factors), connective tissue progenitors rich in stem cells, and augmentation with biological scaffolds. Many studies present both the advantages and the limitations of biological therapies supporting RC repair (Table 1). To improve the healing of the RC, advanced stem cell therapy and gene therapy appear to be the preferred options as they activate the body's potential to repair damaged tissue.

**Table 1:** Advantages and limitations of biological therapies supporting rotator cuff repair.

	Advantages	Limitations
PRP	Autologous nature Low chance of rejection, infection, or system reaction Blood extraction is minimal risk to the patient Readily available Rapid extraction It has no expiration and no shelf life Can be combined with other therapies	No optimal preparation method Lack of evidence regarding long-term follow-up Variable growth factor and cytokine quantities Not cost-effective
Stem Cells	Autologous nature Low chance of rejection, infection, or system reaction Minimal resources required to produce Can be combined with other therapies Cost-effective	Risk of pain during harvest Variable stem cell quantities More time-consuming than PRP
Scaffolds	High hydrophilic properties Low immunological response Cell adhesive Can be combined with other therapies LHB- locally available at no cost	Costly to produce (unless autografts) Shows degradation during long-term storage Variability between donor scaffolds Technically demanding procedure For autografts (LHBT not always present, fascia – donor site morbidity, extra procedure)

Currently, one of the most researched issues is the use of stem cells, modified before implantation by exposure to various growth factors or modification of the culture conditions, to generate the desired phenotype. Moreover, the newly recognized anti-inflammatory and anti-apoptotic effects of stem cells on tissue healing may have enormous potential for recovery and functional restoration. Nevertheless, to make biological treatments more effective and to further improve RC healing, a better understanding of the molecular mechanisms of physiological tendon healing is necessary.

What needs to be strengthened is that most of the studies cited here are limited due to the lack of detailed information on the composition and biological activity of the injected biomaterials. Without the knowledge of these two, the standardization of procedures cannot be obtained, and this is necessary to identify the optimum formulations for different pathologies and different tissues, as well as to obtain reproducible results. In this spirit, in 2017, a consensus on the minimum information for clinical studies evaluating biologics in orthopedics (MIBO) was published (106), so it is now recommended for each publication describing the clinical results of the use of PRP or MSCs to report the cellular composition of whole blood and the delivered PRP, and a robust characterization of the cells being implanted.

A take-home message of this article would be that, since conventional repair of RC tears carries a potential risk of incomplete healing, the biological therapies should be recommended and applied to facilitate regeneration. However, there is a restriction of the need to standardize these procedures and report the characteristics of the material.

## ICMJE Conflict of Interest Statement

The authors declare that there is no conflict of interest that could be perceived as prejudicing the impartiality of the research reported.

## Funding Statement

This work did not receive any specific grant from any funding agency in the public, commercial, or not-for-profit sector.

## References

[1] Prabhakar A Kanthalu Subramanian JN Swathikaa P Kumareswaran SI & Subramanian KN. Current concepts on management of cuff tear. Journal of Clinical Orthopaedics and Trauma 2022 28 101808. (10.1016/j.jcot.2022.101808) 35402155 PMC8983388

[2] Minagawa H Yamamoto N Abe H Fukuda M Seki N Kikuchi K Kijima H & Itoi E. Prevalence of symptomatic and asymptomatic rotator cuff tears in the general population: from mass-screening in one village. Journal of Orthopaedics 2013 10 8–12. (10.1016/j.jor.2013.01.008) 24403741 PMC3768248

[3] Collins T Alexander D & Barkatali B. Platelet-rich plasma: a narrative review. EFORT Open Reviews 2021 6 225–235. (10.1302/2058-5241.6.200017) 34040800 PMC8142058

[4] Yanke A Dandu N Credille K Damodar D Wang Z & Cole BJ. Indications and technique: rotator cuff repair augmentation. Journal of the American Academy of Orthopaedic Surgeons 2023 31 1205–1210. (10.5435/JAAOS-D-23-00101) 37816190

[5] Dierckman BD Frousiakis P Burns JP Barber FA Wodicka R Getelman MH Karzel RP & Snyder SJ. Arthroscopic repair of medium to large rotator cuff tears with a triple-loaded medially based single-row technique augmented with marrow vents. Arthroscopy 2021 37 28–37. (10.1016/j.arthro.2020.08.003) 32805317

[6] Garofalo R De Crescenzo A Fontanarosa A Conti M Castagna A & Calbi R. Rotator cuff repair protected with subacromial balloon spacer shows a low rate of non-healing. Knee Surgery, Sports Traumatology, Arthroscopy 2022 30 2123–2129. (10.1007/s00167-021-06831-1) 35022825

[7] Rashid MS Cooper C Cook J Cooper D Dakin SG Snelling S & Carr AJ. Increasing age and tear size reduce rotator cuff repair healing rate at 1 year. Acta Orthopaedica 2017 88 606–611. (10.1080/17453674.2017.1370844) 28880113 PMC5694804

[8] Galatz LM Ball CM Teefey SA Middleton WD & Yamaguchi K. The outcome and repair integrity of completely arthroscopically repaired large and massive rotator cuff tears. Journal of Bone and Joint Surgery. American Volume 2004 86 219–224. (10.2106/00004623-200402000-00002) 14960664

[9] Franceschi F Ruzzini L Longo UG Martina FM Zobel BB Maffulli N & Denaro V. Equivalent clinical results of arthroscopic single-row and double-row suture anchor repair for rotator cuff tears: a randomized controlled trial. American Journal of Sports Medicine 2007 35 1254–1260. (10.1177/0363546507302218) 17554104

[10] Carpenter JE Thomopoulos S Flanagan CL DeBano CM & Soslowsky LJ. Rotator cuff defect healing: a biomechanical and histologic analysis in an animal model. Journal of Shoulder and Elbow Surgery 1998 7 599–605. (10.1016/s1058-2746(98)90007-6) 9883420

[11] Mandaleson A. Re-tears after rotator cuff repair: current concepts review. Journal of Clinical Orthopaedics and Trauma 2021 19 168–174. (10.1016/j.jcot.2021.05.019) 34123722 PMC8170498

[12] Longo UG Lamberti A Maffulli N & Denaro V. Tissue engineered biological augmentation for tendon healing: a systematic review. British Medical Bulletin 2011 98 31–59. (10.1093/bmb/ldq030) 20851817

[13] Isaac C Gharaibeh B Witt M Wright VJ & Huard J. Biologic approaches to enhance rotator cuff healing after injury. Journal of Shoulder and Elbow Surgery 2012 21 181–190. (10.1016/j.jse.2011.10.004) 22244061

[14] Longo UG Berton A Khan WS Maffulli N & Denaro V. Histopathology of rotator cuff tears. Sports Medicine and Arthroscopy Review 2011 19 227–236. (10.1097/JSA.0b013e318213bccb) 21822106

[15] Longo UG Berton A Papapietro N Maffulli N & Denaro V. Biomechanics of the rotator cuff: European perspective. Medicine and Sport Science 2012 57 10–17. (10.1159/000328870) 21986041

[16] Lafosse L Brzoska R Toussaint B & Gobezie R. The outcome and structural integrity of arthroscopic rotator cuff repair with use of the double-row suture anchor technique: surgical technique. Journal of Bone and Joint Surgery 2008 90(Supplement 2 Pt 2) 275–286. (10.2106/JBJS.H.00388) 18829940

[17] Lui P Zhang P Chan K & Qin L. Biology and augmentation of tendon-bone insertion repair. Journal of Orthopaedic Surgery and Research 2010 21 5–59) 10.1186/1749-799X-5-59 PMC2931497 20727196

[18] Longo UG Franceschi F Ruzzini L Rabitti C Morini S Maffulli N & Denaro V. Characteristics at haematoxylin and eosin staining of ruptures of the long head of the biceps tendon. British Journal of Sports Medicine 2009 43 603–607. (10.1136/bjsm.2007.039016) 18070808

[19] Del Buono A Oliva F Longo UG Rodeo SA Orchard J Denaro V & Maffulli N. Metalloproteases and rotator cuff disease. Journal of Shoulder and Elbow Surgery 2012 21 200–208. (10.1016/j.jse.2011.10.020) 22244063

[20] Fabiś J Szemraj J Strek M Fabiś A Dutkiewicz Z & Zwierzchowski TJ. Is resection of the tendon edge necessary to enhance the healing process? An evaluation of the homeostasis of apoptotic and inflammatory processes in the distal 1 cm of a torn supraspinatus tendon: Part I. Journal of Shoulder and Elbow Surgery 2014 23 1772–1778. (10.1016/j.jse.2014.03.018) 24927882

[21] Lin KM Frey CS Atzmon R Pierre K Vel MS & Sherman SL. Orthobiologic techniques for surgical augmentation. Physical Medicine and Rehabilitation Clinics of North America 2023 34 265–274. (10.1016/j.pmr.2022.08.015) 36410886

[22] Bono OJ Jenkin B Forlizzi J Mousad A Le Breton S MacAskill M Mandalia K Mithoefer K Ramappa A Ross G *et al.* Evidence for utilization of injectable biologic augmentation in primary rotator cuff repair: a systematic review of data from 2010 to 2022. Orthopaedic Journal of Sports Medicine 2023 11 23259671221150037. (10.1177/23259671221150037) 36756167 PMC9900676

[23] Weibrich G Kleis WKG Hafner G & Hitzler WE. Growth factor levels in platelet-rich plasma and correlations with donor age, sex, and platelet count. Journal of Cranio-Maxillo-Facial Surgery 2002 30 97–102. (10.1054/jcms.2002.0285) 12069512

[24] Smith CW. Release of alpha-granule contents during platelet activation. Platelets 2022 33 491–502. (10.1080/09537104.2021.1913576) 34569425

[25] Alves R & Grimalt R. A review of platelet-rich plasma: history, biology, mechanism of action, and classification. Skin Appendage Disorders 2018 4 18–24. (10.1159/000477353) 29457008 PMC5806188

[26] Mizuno M Katano H Otabe K Komori K Matsumoto Y Fujii S Ozeki N Tsuji K Koga H Muneta T *et al.* Platelet-derived growth factor (PDGF)-AA/AB in human serum are potential indicators of the proliferative capacity of human synovial mesenchymal stem cells. Stem Cell Research and Therapy 2015 6 243. (10.1186/s13287-015-0239-2) 26652649 PMC4675012

[27] Klein GL. Transforming growth factor-beta in skeletal muscle wasting. International Journal of Molecular Sciences 2022 23 1167. (10.3390/ijms23031167) 35163088 PMC8835446

[28] Shaw P Dwivedi SKD Bhattacharya R Mukherjee P & Rao G. VEGF signaling: role in angiogenesis and beyond. Biochimica et Biophysica Acta (BBA) – Reviews on Cancer 2024 1879 189079. (doi:10.1016/j.bbcan.2024.189079. 38280470 10.1016/j.bbcan.2024.189079 PMC12927493

[29] Snow M Hussain F Pagkalos J Kowalski T Green M Massoud S & James S. The effect of delayed injection of leukocyte-rich platelet-rich plasma following rotator cuff repair on patient function: A randomized double-blind controlled trial. Arthroscopy 2020 36 648–657. (10.1016/j.arthro.2019.09.026) 31784365

[30] Walsh MR Nelson BJ Braman JP Yonke B Obermeier M Raja A & Reams M. Platelet-rich plasma in fibrin matrix to augment rotator cuff repair: a prospective, single-blinded, randomized study with 2-year follow-up. Journal of Shoulder and Elbow Surgery 2018 27 1553–1563. (10.1016/j.jse.2018.05.003) 29996980

[31] Gurger M Once G Yilmaz E Demir S Calik I Say Y Kavakli A Key S Gurbuz MU & Bingollu O. The effect of the platelet-rich plasma and ozone therapy on tendon-to-bone healing in the rabbit rotator cuff repair model. Journal of Orthopaedic Surgery and Research 2021 16 202. (10.1186/s13018-021-02320-w) 33740995 PMC7976715

[32] Cai YU Sun Z Liao B Song Z Xiao T & Zhu P. Sodium hyaluronate and platelet-rich plasma for partial-thickness rotator cuff tears. Medicine and Science in Sports and Exercise 2019 51 227–233. (10.1249/MSS.0000000000001781) 30199423 PMC6336488

[33] Malavolta EA Gracitelli MEC Assunção JH Ferreira Neto AA Bordalo-Rodrigues M & de Camargo OP. Clinical and structural evaluations of rotator cuff repair with and without added platelet-rich plasma at 5-year follow-up: a prospective randomized study. American Journal of Sports Medicine 2018 46 3134–3141. (10.1177/0363546518795895) 30234999

[34] Ahmad Z Ang S Rushton N Harvey A Akhtar K Dawson-Bowling S & Noorani A. Platelet-rich plasma augmentation of arthroscopic rotator cuff repair lowers retear rates and improves short-term postoperative functional outcome scores: a systematic review of meta-analyses. Arthroscopy, Sports Medicine, and Rehabilitation 2022 4 e823–e833. (10.1016/j.asmr.2021.12.012) 35494273 PMC9042896

[35] Warth RJ Dornan GJ James EW Horan MP & Millett PJ. Clinical and structural outcomes after arthroscopic repair of full-thickness rotator cuff tears with and without platelet-rich product supplementation: a meta-analysis and meta-regression. Arthroscopy 2015 31 306–320. (10.1016/j.arthro.2014.09.007) 25450417

[36] Zhang Z Wang Y & Sun J. The effect of platelet-rich plasma on arthroscopic double-row rotator cuff repair: a clinical study with 12-month follow-up. Acta Orthopaedica et Traumatologica Turcica 2016 50 191–197. (10.3944/AOTT.2015.15.0113) 26969955

[37] Vavken P Sadoghi P Palmer M Rosso C Mueller AM Szoelloesy G & Valderrabano V. Platelet-rich plasma reduces retear rates after arthroscopic repair of small- and medium-sized rotator cuff tears but is not cost-effective. American Journal of Sports Medicine 2015 43 3071–3076. (10.1177/0363546515572777) 25767267

[38] Cai YZ Zhang C & Lin XJ. Efficacy of platelet-rich plasma in arthroscopic repair of full-thickness rotator cuff tears: a meta-analysis. Journal of Shoulder and Elbow Surgery 2015 24 1852–1859. (10.1016/j.jse.2015.07.035) 26456434

[39] Peng Y Guanglan W Jia S & Zheng C. Leukocyte-rich and leukocyte-poor platelet-rich plasma in rotator cuff repair: A meta-analysis. International Journal of Sports Medicine 2022 43 921–930. (10.1055/a-1790-7982) 35255508

[40] Miranda I Sánchez-Alepuz E Lucas FJ Carratalá V & González-Jofre CA. Use of platelet-rich plasma in the treatment of rotator cuff pathology. What has been scientifically proven? Revista Española de Cirugía Ortopédica y Traumatología 2017 61 249–258. (10.1016/j.recot.2017.03.001) 28529030

[41] Lin MT Wei KC & Wu CH. Effectiveness of platelet-rich plasma injection in rotator cuff tendinopathy: a systematic review and meta-analysis of randomized controlled trials. Diagnostics 2020 10 189. (10.3390/diagnostics10040189) 32231127 PMC7235747

[42] Velasquez Garcia A Ingala Martini L Franco Abache A & Abdo G. Role of platelet-rich plasma in the treatment of rotator cuff tendinopathy. World Journal of Orthopedics 2023 14 505–515. (10.5312/wjo.v14.i7.505) 37485430 PMC10359750

[43] Samuelson EM Odum SM & Fleischli JE. The cost-effectiveness of using platelet-rich plasma during rotator cuff repair: a markov model analysis. Arthroscopy 2016 32 1237–1244. (10.1016/j.arthro.2015.12.018) 26927681

[44] Longo UG Carnevale A Piergentili I Berton A Candela V Schena E & Denaro V. Retear rates after rotator cuff surgery: a systematic review and meta-analysis. BMC Musculoskeletal Disorders 2021 22 749. (10.1186/s12891-021-04634-6) 34465332 PMC8408924

[45] Liu J Gao J Liang Z Gao C Niu Q Wu F & Zhang L. Mesenchymal stem cells and their microenvironment. Stem Cell Research and Therapy 2022 13 429. (10.1186/s13287-022-02985-y) 35987711 PMC9391632

[46] Costela-Ruiz VJ Melguizo-Rodríguez L Bellotti C Illescas-Montes R Stanco D Arciola CR & Lucarelli E. Different sources of mesenchymal stem cells for tissue regeneration: a guide to identifying the most favorable one in orthopedics and dentistry applications. International Journal of Molecular Sciences 2022 23 6356. (10.3390/ijms23116356) 35683035 PMC9181542

[47] Furia JP Lundeen MA Hurd JL Pearce DA Alt C Alt EU Schmitz C & Maffulli N. Why and how to use the body’s own stem cells for regeneration in musculoskeletal disorders: a primer. Journal of Orthopaedic Surgery and Research 2022 17 36. (10.1186/s13018-022-02918-8) 35062984 PMC8781360

[48] Wang HN Rong X Yang LM Hua WZ & Ni GX. Advances in stem cell therapies for rotator cuff injuries. Frontiers in Bioengineering and Biotechnology 2022 10 866195. (10.3389/fbioe.2022.866195) 35694228 PMC9174670

[49] Gonzalez-Vilchis RA Piedra-Ramirez A Patiño-Morales CC Sanchez-Gomez C & Beltran-Vargas NE. Sources, characteristics, and therapeutic applications of mesenchymal cells in tissue engineering. Tissue Engineering and Regenerative Medicine 2022 19 325–361. (10.1007/s13770-021-00417-1) 35092596 PMC8971271

[50] Milano G Saccomanno MF Careri S Taccardo G De Vitis R & Fabbriciani C. Efficacy of marrow-stimulating technique in arthroscopic rotator cuff repair: a prospective randomized study. Arthroscopy 2013 29 802–810. (10.1016/j.arthro.2013.01.019) 23522987

[51] Taniguchi N Suenaga N Oizumi N Miyoshi N Yamaguchi H Inoue K & Chosa E. Bone marrow stimulation at the footprint of arthroscopic surface-holding repair advances cuff repair integrity. Journal of Shoulder and Elbow Surgery 2015 24 860–866. (10.1016/j.jse.2014.09.031) 25487905

[52] Toro F Pinochet F Ruiz F Moraga C Pozo R Oliva JP Reinares F & Mardones P. Functional and radiological results of the crimson duvet procedure in rotator cuff treatment: a randomize controlled clinical trial. Journal of Shoulder and Elbow Surgery 2022 31 1200–1207. (10.1016/j.jse.2021.12.004) 35007748

[53] Zhang L Zhu Y Xu T & Fu W. Bone marrow stimulation in arthroscopic rotator cuff repair is a cost-effective and straightforward technique to reduce retear rates: a systematic review and meta-analysis. Frontiers in Surgery 2023 10 1047483. (10.3389/fsurg.2023.1047483) 36896263 PMC9989271

[54] Osti L Del Buono A & Maffulli N. Microfractures at the rotator cuff footprint: a randomised controlled study. International Orthopaedics 2013 37 2165–2171. (10.1007/s00264-013-1952-z) 23760681 PMC3824902

[55] Kida Y Morihara T Matsuda KI Kajikawa Y Tachiiri H Iwata Y Sawamura K Yoshida A Oshima Y Ikeda T *et al.* Bone marrow-derived cells from the footprint infiltrate into the repaired rotator cuff. Journal of Shoulder and Elbow Surgery 2013 22 197–205. (10.1016/j.jse.2012.02.007) 22543003

[56] Bilsel K Yildiz F Kapicioglu M Uzer G Elmadag M Pulatkan A Esrefoglu M Bozdag E & Milano G. Efficacy of bone marrow-stimulating technique in rotator cuff repair. Journal of Shoulder and Elbow Surgery 2017 26 1360–1366. (10.1016/j.jse.2017.02.014) 28395947

[57] Shin KH Kim JU Jang IT & Han SB. Effect of bone marrow stimulation on arthroscopic rotator cuff repair: a systematic review and meta-analysis. Orthopaedic Journal of Sports Medicine 2024 12 23259671231224482. (10.1177/23259671231224482) 38282788 PMC10812110

[58] Le Breton S Forlizzi J Bono O MacAskill M Mousad A Kush S O'Brien M Christensen A Mithoefer K Ramappa A *et al.* Local intraoperative marrow-derived augmentation for primary rotator cuff repair: an updated systematic review and meta-analysis of studies from 2010 to 2022. Orthopaedic Journal of Sports Medicine 2023 11 23259671221147896. (10.1177/23259671221147896) 37009491 PMC10061649

[59] Ajrawat P Dwyer T Almasri M Veillette C Romeo A Leroux T Theodoropoulos J Nauth A Henry P & Chahal J. Bone marrow stimulation decreases retear rates after primary arthroscopic rotator cuff repair: a systematic review and meta-analysis. Journal of Shoulder and Elbow Surgery 2019 28 782–791. (10.1016/j.jse.2018.11.049) 30885313

[60] Hegde V Shonuga O Ellis S Fragomen A Kennedy J Kudryashov V & Lane JM. A prospective comparison of 3 approved systems for autologous bone marrow concentration demonstrated nonequivalency in progenitor cell number and concentration. Journal of Orthopaedic Trauma 2014 28 591–598. (10.1097/BOT.0000000000000113) 24694554

[61] Murphy EP Fenelon C McGoldrick NP & Kearns SR. Bone marrow aspirate concentrate and microfracture technique for talar osteochondral lesions of the ankle. Arthroscopy Techniques 2018 7 e391–e396. (10.1016/j.eats.2017.10.011) 29868410 PMC5982938

[62] Gulotta LV Kovacevic D Ehteshami JR Dagher E Packer JD & Rodeo SA. Application of bone marrow-derived mesenchymal stem cells in a rotator cuff repair model. American Journal of Sports Medicine 2009 37 2126–2133. (10.1177/0363546509339582) 19684297

[63] Gulotta LV Kovacevic D Montgomery S Ehteshami JR Packer JD & Rodeo SA. Stem cells genetically modified with the developmental gene MT1-MMP improve regeneration of the supraspinatus tendon-to-bone insertion site. American Journal of Sports Medicine 2010 38 1429–1437. (10.1177/0363546510361235) 20400753

[64] Gulotta LV Kovacevic D Packer JD Deng XH & Rodeo SA. Bone marrow-derived mesenchymal stem cells transduced with scleraxis improve rotator cuff healing in a rat model. American Journal of Sports Medicine 2011 39 1282–1289. (10.1177/0363546510395485) 21335341

[65] Hernigou P Flouzat Lachaniette CH Delambre J Zilber S Duffiet P Chevallier N & Rouard H. Biologic augmentation of rotator cuff repair with mesenchymal stem cells during arthroscopy improves healing and prevents further tears: a case-controlled study. International Orthopaedics 2014 38 1811–1818. (10.1007/s00264-014-2391-1) 24913770

[66] Ellera Gomes JL da Silva RC Silla LMR Abreu MR & Pellanda R. Conventional rotator cuff repair complemented by the aid of mononuclear autologous stem cells. Knee Surgery, Sports Traumatology, Arthroscopy 2012 20 373–377. (10.1007/s00167-011-1607-9) PMC3262133 21773831

[67] Cole BJ Kaiser JT Wagner KR Sivasundaram L Otte RS Tauro TM White GM Ralls ML Yanke AB Forsythe B *et al.* Prospective randomized trial of biologic augmentation with bone marrow aspirate concentrate in patients undergoing arthroscopic rotator cuff repair. American Journal of Sports Medicine 2023 51 1234–1242. (10.1177/03635465231154601) 36811557

[68] Bunnell BA. Adipose tissue-derived mesenchymal stem cells. Cells 2021 10 3433. (10.3390/cells10123433) 34943941 PMC8700397

[69] Kokubu S Inaki R Hoshi K & Hikita A. Adipose-derived stem cells improve tendon repair and prevent ectopic ossification in tendinopathy by inhibiting inflammation and inducing neovascularization in the early stage of tendon healing. Regenerative Therapy 2020 14 103–110. (10.1016/j.reth.2019.12.003) 31989000 PMC6970144

[70] Wang C Zhang Y Zhang G Yu W & He Y. Adipose stem cell-derived exosomes ameliorate chronic rotator cuff tendinopathy by regulating macrophage polarization: from a mouse model to a study in human tissue. American Journal of Sports Medicine 2021 49 2321–2331. (10.1177/03635465211020010) 34259608

[71] Oh JH Chung SW Kim SH Chung JY & Kim JY. 2013 Neer Award: effect of the adipose-derived stem cell for the improvement of fatty degeneration and rotator cuff healing in rabbit model. Journal of Shoulder and Elbow Surgery 2014 23 445–455. (10.1016/j.jse.2013.07.054) 24129058

[72] Valencia Mora M Antuña Antuña S García Arranz M Carrascal MT & Barco R. Application of adipose tissue-derived stem cells in a rat rotator cuff repair model. Injury 2014 45(Supplement 4) S22–S27. (10.1016/S0020-1383(14)70006-3) 25384471

[73] Kim YS Sung CH Chung SH Kwak SJ & Koh YG. Does an injection of adipose-derived mesenchymal stem cells loaded in fibrin glue influence rotator cuff repair outcomes? A clinical and magnetic resonance imaging study. American Journal of Sports Medicine 2017 45 2010–2018. (10.1177/0363546517702863) 28448728

[74] Jo CH Chai JW Jeong EC Oh S & Yoon KS. Intratendinous injection of mesenchymal stem cells for the treatment of rotator cuff disease: a 2-year follow-up study. Arthroscopy 2020 36 971–980. (10.1016/j.arthro.2019.11.120) 31805388

[75] Hurd JL Facile TR Weiss J Hayes M Hayes M Furia JP Maffulli N Winnier GE Alt C Schmitz C *et al.* Safety and efficacy of treating symptomatic, partial-thickness rotator cuff tears with fresh, uncultured, unmodified, autologous adipose-derived regenerative cells (UA-ADRCs) isolated at the point of care: a prospective, randomized, controlled first-in-human pilot study. Journal of Orthopaedic Surgery and Research 2020 15 122. (10.1186/s13018-020-01631-8) 32238172 PMC7110715

[76] Lipner J Shen H Cavinatto L Liu W Havlioglu N Xia Y Galatz LM & Thomopoulos S. In vivo evaluation of adipose-derived stromal cells delivered with a nanofiber scaffold for tendon-to-bone repair. Tissue Engineering. Part A 2015 21 2766–2774. (10.1089/ten.TEA.2015.0101) 26414599 PMC4652200

[77] Kaizawa Y Franklin A Leyden J Behn AW Tulu US Sotelo Leon D Wang Z Abrams GD Chang J & Fox PM. Augmentation of chronic rotator cuff healing using adipose-derived stem cell-seeded human tendon-derived hydrogel. Journal of Orthopaedic Research 2019 37 877–886. (10.1002/jor.24250) 30747435

[78] Rothrauff BB Smith CA Ferrer GA Novaretti JV Pauyo T Chao T Hirsch D Beaudry MF Herbst E Tuan RS *et al.* The effect of adipose-derived stem cells on enthesis healing after repair of acute and chronic massive rotator cuff tears in rats. Journal of Shoulder and Elbow Surgery 2019 28 654–664. (10.1016/j.jse.2018.08.044) 30527883

[79] Shin MJ Shim IK Kim DM Choi JH Lee YN Jeon IH Kim H Park D Kholinne E Yang HS *et al.* Engineered cell sheets for the effective delivery of adipose-derived stem cells for tendon-to-bone healing. American Journal of Sports Medicine 2020 48 3347–3358. (10.1177/0363546520964445) 33136454

[80] Randelli P Menon A Ragone V Creo P Bergante S Randelli F De Girolamo L Alfieri Montrasio U Banfi G Cabitza P *et al.* Lipogems product treatment increases the proliferation rate of human tendon stem cells without affecting their stemness and differentiation capability. Stem Cells International 2016 2016 4373410. (10.1155/2016/4373410) 27057170 PMC4736573

[81] Klatte-Schulz F Thiele K Scheibel M Duda GN & Wildemann B. Subacromial bursa: a neglected tissue is gaining more and more attention in clinical and experimental research. Cells 2022 11 663. (10.3390/cells11040663) 35203311 PMC8870132

[82] Muench LN Baldino JB Berthold DP Kia C Lebaschi A Cote MP McCarthy MB & Mazzocca AD. Subacromial bursa-derived cells demonstrate high proliferation potential regardless of patient demographics and rotator cuff tear characteristics. Arthroscopy 2020 36 2794–2802. (10.1016/j.arthro.2020.06.008) 32554077

[83] Kriscenski DE Lebaschi A Tamburini LM McCarthy MBR Cote MP Kumbar SG & Mazzocca AD. Characterization of murine subacromial bursal-derived cells. Connective Tissue Research 2022 63 287–297. (10.1080/03008207.2021.1917556) 34042553

[84] Morikawa D LeVasseur MR Luczak SB Mancini MR Bellas N McCarthy MBR Cote MP Berthold DP Muench LN & Mazzocca AD. Decreased colony-forming ability of subacromial bursa-derived cells during revision arthroscopic rotator cuff repair. Arthroscopy, Sports Medicine, and Rehabilitation 2021 3 e1047–e1054. (10.1016/j.asmr.2021.03.010) 34430884 PMC8365201

[85] Xia H Liang C Luo P Huang J He J Wang Z Cao X Peng C & Wu S. Pericellular collagen I coating for enhanced homing and chondrogenic differentiation of mesenchymal stem cells in direct intra-articular injection. Stem Cell Research and Therapy 2018 9 174. (10.1186/s13287-018-0916-z) 29945671 PMC6020325

[86] Enomoto T Akagi R Ogawa Y Yamaguchi S Hoshi H Sasaki T Sato Y Nakagawa R Kimura S Ohtori S *et al.* Timing of intra-articular injection of synovial mesenchymal stem cells affects cartilage restoration in a partial thickness cartilage defect model in rats. Cartilage 2020 11 122–129. (10.1177/1947603518786542) 29989441 PMC6921951

[87] Karuppaiah K & Sinha J. Scaffolds in the management of massive rotator cuff tears: current concepts and literature review. EFORT Open Reviews 2019 4 557–566. (10.1302/2058-5241.4.180040) 31598334 PMC6771075

[88] Ozaki J Fujimoto S Masuhara K Tamai S & Yoshimoto S. Reconstruction of chronic massive rotator cuff tears with synthetic materials. Clinical Orthopaedics and Related Research 1986 (202) 173–183. (10.1097/00003086-198601000-00022) 3955946

[89] Barth J Olmos MI Swan J Barthelemy R Delsol P & Boutsiadis A. Superior capsular reconstruction with the long head of the biceps autograft prevents infraspinatus retear in massive posterosuperior retracted rotator cuff tears. American Journal of Sports Medicine 2020 48 1430–1438. (10.1177/0363546520912220) 32267730

[90] Brzóska R Laprus H Michniowski P Solecki W Klon W & Błasiak A. Novel and effective arthroscopic extracapsular stabilization technique for anterior shoulder instability-BLS. Knee Surgery, Sports Traumatology, Arthroscopy 2019 27 3897–3904. (10.1007/s00167-019-05496-1) 30941470

[91] Veen EJD Stevens M & Diercks RL. Biceps autograft augmentation for rotator cuff repair: a systematic review. Arthroscopy 2018 34 1297–1305. (10.1016/j.arthro.2017.10.044) 29373293

[92] Baker AR McCarron JA Tan CD Iannotti JP & Derwin KA. Does augmentation with a reinforced fascia patch improve rotator cuff repair outcomes? Clinical Orthopaedics and Related Research 2012 470 2513–2521. (10.1007/s11999-012-2348-x) 22528381 PMC3830110

[93] Malcarney HL Bonar F & Murrell GAC. Early inflammatory reaction after rotator cuff repair with a porcine small intestine submucosal implant: a report of 4 cases. American Journal of Sports Medicine 2005 33 907–911. (10.1177/0363546504271500) 15827358

[94] Iannotti JP Codsi MJ Kwon YW Derwin K Ciccone J & Brems JJ. Porcine small intestine submucosa augmentation of surgical repair of chronic two-tendon rotator cuff tears. A randomized, controlled trial. Journal of Bone and Joint Surgery 2006 88 1238–1244. (10.2106/JBJS.E.00524) 16757756

[95] Walton JR Bowman NK Khatib Y Linklater J & Murrell GAC. Restore orthobiologic implant: not recommended for augmentation of rotator cuff repairs. Journal of Bone and Joint Surgery 2007 89 786–791. (10.2106/JBJS.F.00315) 17403801

[96] Bokor DJ Sonnabend D Deady L Cass B Young A Van Kampen C & Arnoczky S. Preliminary investigation of a biological augmentation of rotator cuff repairs using a collagen implant: a 2-year MRI follow-up. Muscles, Ligaments and Tendons Journal 2015 5 144–150. (10.11138/mltj/2015.5.3.144) 26605186 PMC4617212

[97] Bokor DJ Sonnabend DH Deady L Cass B Young AA Van Kampen CL & Arnoczky SP. Healing of partial-thickness rotator cuff tears following arthroscopic augmentation with a highly porous collagen implant: a 5-year clinical and MRI follow-up. Muscle Ligaments and Tendons Journal 2019 09 338–347. (10.32098/mltj.03.2019.07) PMC4915456 27331028

[98] Bushnell BD Connor PM Harris HW Ho CP Trenhaile SW & Abrams JS. Two-year outcomes with a bioinductive collagen implant used in augmentation of arthroscopic repair of full-thickness rotator cuff tears: final results of a prospective multi-center study. Journal of Shoulder and Elbow Surgery 2022 31 2532–2541. (10.1016/j.jse.2022.05.025) 35788057

[99] McIntyre LF Bishai SK Brown PB Bushnell BD & Trenhaile SW. Patient-reported outcomes following use of a bioabsorbable collagen implant to treat partial and full-thickness rotator cuff tears. Arthroscopy 2019 35 2262–2271. (10.1016/j.arthro.2019.02.019) 31350082

[100] Goldenberg BT Lacheta L Dekker TJ Spratt JD Nolte PC & Millett PJ. Biologics to improve healing in large and massive rotator cuff tears: a critical review. Orthopedic Research and Reviews 2020 12 151–160. (10.2147/ORR.S260657) 33116954 PMC7568683

[101] Condron NB Kester BS Tokish JM Zumstein MA Gobezie R Scheibel M & Cole BJ. Nonoperative and operative soft-tissue, cartilage, and bony regeneration and orthopaedic biologics of the shoulder: an orthoregeneration network (ON) foundation review. Arthroscopy 2021 37 3200–3218. (10.1016/j.arthro.2021.06.033) 34293441

[102] Zhang C Wu J Li X Wang Z Lu WW & Wong TM. Current biological strategies to enhance surgical treatment for rotator cuff repair. Frontiers in Bioengineering and Biotechnology 2021 9 657584. (10.3389/fbioe.2021.657584) 34178957 PMC8226184

[103] Bailey JR Kim C Alentorn-Geli E Kirkendall DT Ledbetter L Taylor DC Toth AP & Garrigues GE. Rotator cuff matrix augmentation and interposition: A systematic review and meta-analysis. American Journal of Sports Medicine 2019 47 1496–1506. (10.1177/0363546518774762) 29906191

[104] Baldwin M Nagra NS Greenall G Carr AJ Beard D Rees JL Rangan A Merritt N Dritsaki M Hopewell S *et al.* Use of implantable meshes for augmented rotator cuff repair: a systematic review and meta-analysis. BMJ Open 2020 10 e039552. (10.1136/bmjopen-2020-039552) PMC7722806 33293307

[105] Baldwin MJ Nagra NS Merritt N Rees JL Carr AJ Rangan A Thomas M Beard DJ Cooper C Kottam L *et al.* The use of a patch to augment rotator cuff surgery - A survey of UK shoulder and elbow surgeons. PLoS One 2020 15 e0230235. (10.1371/journal.pone.0230235) 32240199 PMC7117708

[106] Murray IR Geeslin AG Goudie EB Petrigliano FA & LaPrade RF. Minimum information for studies evaluating biologics in orthopaedics (MIBO): platelet-rich plasma and mesenchymal stem cells. Journal of Bone and Joint Surgery 2017 99 809–819. (10.2106/JBJS.16.00793) 28509821

